# Design of Magnetic Fluid-Enhanced Optical Fiber Polarization Filter

**DOI:** 10.3390/mi15111364

**Published:** 2024-11-11

**Authors:** Haixu Chen, Lianzhen Zhang, Xin Ding

**Affiliations:** 1 College of Energy Enginrering, Huanghuai University, Zhumadian 463000, China; chenhaixu@huanghuai.edu.cn; 2 College of Automotive Engineering, Shandong Polytechnic College, Jining 272067, China; 3 Key Laboratory of Optoelectronic Devices and Systems of Ministry of Education/Guangdong Province, College of Physics and Optoelectronic Engineering, Shenzhen University, Shenzhen 518060, China; danielxin2019@126.com

**Keywords:** fiber polarization filter, surface plasmon resonance, magnetic fluid, photonic crystal fiber

## Abstract

In this paper, we demonstrated a method of filling the air holes of a photonic crystal fiber (PCF), coated with gold film, with magnetic fluid (MF) to enhance the Surface Plasmon Resonance (SPR). The simulation results show that at the wavelength of 1260–1675 nm, the minimum loss coefficient of the y-polarization mode is 4.7 times that before filling with MF, and the x-polarization mode is 0.45 times greater. Then, based on this method, we designed a polarizing filter with a core diameter of 9 µm. The numerical simulation results indicate that it not only maintains the same core diameter as the single-mode fiber, but also has a larger bandwidth and a higher extinction ratio (ER). Additionally, we can optimize its ER at a specific wavelength by adjusting the magnetic field.

## 1. Introduction

Polarization filters act as polarizers in the polarization manipulation process, commonly used in fiber optic gyroscope systems, polarization-maintaining fiber amplifiers, coherent optical communication systems, and information security optical systems [1,2,3]. With the continuous development of micro-machining technology, metals and optical fibers can be combined to introduce Surface Plasmon Resonance (SPR) [4]. Utilizing the SPR effect can significantly improve its working characteristics, such as polarization ER, operating bandwidth, etc. [5,6,7,8,9,10,11]. All-fiber polarization filters reported in recent years mainly include the following ways of combining optical fibers with metal. The first method is to fill the air holes of photonic crystal fibers (PCFs) with metal wires or coat them with metal films [12,13,14,15,16,17,18,19,20,21,22]. Another way is to make the optical fiber into a D-shape and coat its surface with a metal film [23,24,25,26,27,28,29].

However, polarization filters that coat the metal thin films in the air holes of a PCF reduce the core diameter in order to enhance the performance of the fiber polarization filter, which will lead to an increased splicing loss with the traditional single-mode optical fiber due to the diameter mismatch. To reduce the loss, it is necessary to explore new methods for improving the performance of the polarization filter without reducing the core diameter. In the past few years, some new methods have been proposed, such as Indra et al. proposing to conduct the nanopatterning of asymmetric metallic metasurface arrays on the end facets of fibers [30]. Although this method does not need to consider the core diameter, it will make the polarization filter unable to be fused with the optical fiber. Zhuo et al. came up with a two-dimensional material-enhanced optical fiber polarization filter [31]. The single-mode optical fiber was first side-polished, and then Au and graphene/MoS2 were integrated onto the polished surface. Two-dimensional (2D) materials can enhance the performance of polarization filters and make their properties electrically tunable. However, the ER and bandwidth obtained by this method are not excellent.

As we all know, the main factor affecting fiber splicing loss is the consistency of the cores. To reduce its impact on splicing loss, it is necessary to find more effective ways to achieve a higher ER and a larger operating bandwidth without reducing the diameter. Here, we propose a method to enhance the SPR by filling MF into the air holes coated with gold. Numerical analysis indicates that it could enhance the SPR of the y-polarization mode while reducing the x-direction. Based on this, we designed a polarization filter with a core diameter of 9 µm, which has a high ER in the wavelength of 1260~1675 nm. In addition, because MF has magnetic sensitivity, the polarization filter characteristics at specific wavelengths can be adjusted by changing the magnetic field.

## 2. Model and Theory

**Figure 1:**
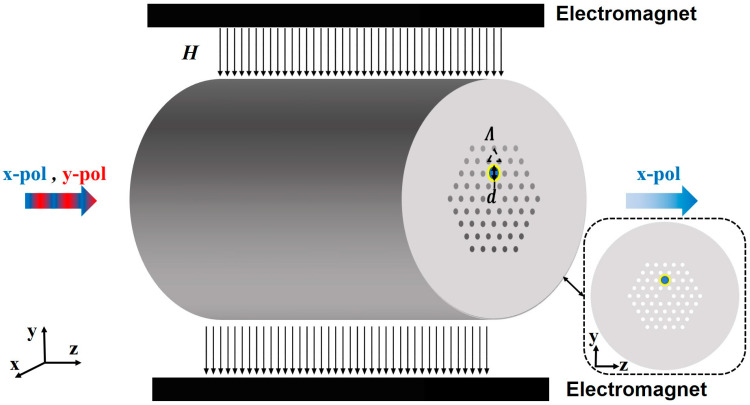
Schematic illustration and cross section of PCF polarization filter based on magnetic fluid enhancement.

Figure 1 shows the schematic diagram of the PCF polarization filter based on magnetic fluid enhancement. The cladding is composed of air holes arranged periodically in an equilateral triangle with a period of *Λ* = 5.25 µm, and the core is formed by replacing the middle air holes with SiO_2_ solid rods. This not only allows the fiber core to possess a diameter comparable to that of single-mode optical fibers, but also ensures adequate space for the creation of suitably sized air holes coated with a gold thin film. To ensure the infinite cutoff single-mode characteristic of the PCF, the duty cycle is selected as 0.44. A gold film with a thickness of *s* = 30 nm is deposited on the inner walls of the air holes with an outer diameter of *d* = 7 µm above the fiber core, as shown in the yellow ring part.

The base material of the PCF is silica, and its dispersion model can be described by the Sellmeier equation [32]. The dielectric constant composite Drude–Lorentz model of gold [33] is expressed as follows:

(1) $$\varepsilon_{r}\left(\omega \right)=1-\frac{\Omega_{p}^{2}}{\omega \left(\omega -i\Gamma_{0}\right)}+\sum_{j=1}^{k}\frac{f_{j}\omega_{p}^{2}}{\left(\omega_{j}^{2}-\omega^{2}\right)+i\omega \Gamma_{j}}$$

where $\omega_{p}$ is the plasma frequency, $\Omega_{p}=\sqrt{f_{0}}\omega_{p}$ represents the plasma frequency associated with the intra-band transmission, $f_{0}$ is the intra-band transmission oscillation intensity, k is the inter-band transmission frequency, $f_{i}$ is the intensity, $1/\Gamma_{j}$ is the lifetime, and $\Gamma_{0}$ represents the attenuation constant. Their values are as follows: $\hbar \omega_{p}=9.03\;\mathrm{eV}$, $\Gamma_{0}=0.048\;\mathrm{eV}$, $f_{0}=0.845$, k=5, $f_{1}=0.065$, $f_{2}=0.124$, $f_{3}=0.011$, $f_{4}=0.840$, $f_{5}=0.5646$, $\Gamma_{1}=3.886\;\mathrm{eV}$, $\Gamma_{2}=0.452\;\mathrm{eV}$, $\Gamma_{3}=8.185\;\mathrm{eV}$, $\Gamma_{4}=0.916\;\mathrm{eV}$, $\Gamma_{5}=2.419\;\mathrm{eV}$, $\omega_{1}=0.816\;\mathrm{eV}$, $\omega_{2}=4.481\;\mathrm{eV}$, $\omega_{3}=8.185\;\mathrm{eV}$, $\omega_{4}=9.083\;\mathrm{eV}$, and $\omega_{5}=20.29\;\mathrm{eV}$.

The air holes coated with the gold film are filled with MF, as shown in the blue part in Figure 1. The variation in the refractive index of the MF with the magnetic field strength can be described by the Langevin function [34,35].

(2) $$n_{\mathrm{MF}}=\left(n_{s}-n_{o}\right)\left[\coth\left(\alpha \frac{H-H_{c}}{T}\right)-\frac{T}{\alpha \left(H-H_{c}\right)}\right]+n_{o}$$

where $n_{o}$ is the refractive index of the MF when the ambient magnetic field intensity is less than $H_{c}$ and its value is determined by the concentration of magnetic particles and the type of base fluid, and $n_{s}$ is the saturation refractive index of the MF. *H* and *T* are the magnetic field strength (in unit Oe; 1 Oe = 79.5775 A·m^−1^) and temperature (in unit K) of the environment, respectively, and α represents the fitting parameters. Here, we use a water-based MF with a particle volume concentration of 0.53 emu/g; the critical magnetic field and ambient temperature are 43 Oe and 294 K, and its $n_{o}$ and $n_{s}$ are 1.4395 and 1.444, respectively. The absorption coefficients α of the fiber polarization can be calculated by Formula (3).

(3) αloss=40πln10Imneff/λdB/μm

where Im(neff) is the imaginary part of the effective refractive index. The extinction ratio (ER) of the x- and y-polarization modes is an important parameter for measuring the performance of the optical fiber polarization filter, and its calculation method is shown in Formula (4).

(4) $$ER=20\mathrm{lg}\left(e^{\left(\alpha_{\mathrm{loss}1}-\alpha_{\mathrm{loss}1}\right)L}\right)$$

where $\alpha_{\mathrm{loss}1}$ and $\alpha_{\mathrm{loss}2}$ represent the unit loss values of the high-loss and low-loss polarization modes, respectively, and *L* represents the length of the optical fiber polarization filter.

## 3. Results and Analysis

**Figure 2:**
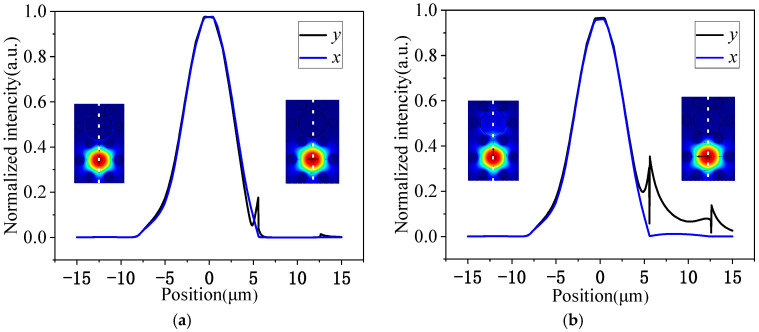
The normalized field intensities of the x- and y-polarization modes at a wavelength of 1550 nm, (**a**) unfilled with MF and (**b**) filled with MF.

Filling the air holes coated with gold film with MF will affect the phase matching point between the core mode and the SPPs mode, thus affecting the polarization filtering performance of the device. Here, the finite element method (FEM) is used to analyze the specific impact of the MF filling on the device filtering performance. Figure 2 shows the electric field distribution in the PCF, either filled with MF or not. The curve in the figure represents the normalized field intensity along the white line in the inset. Figure 2a describes the normalized field intensities of the x- and y-polarization modes at a wavelength of 1550 nm without MF, and Figure 2b depicts the simulation results after filling with MF. The simulation results show that the normalized intensity of the y-polarization mode is more than twice that of the unfilled MF, while the x-polarization mode remains almost unchanged. So, it can be seen that the absorption of the Au film in the y-polarization mode is enhanced by the MF filling at the wavelength of 1550 nm.

**Figure 3:**
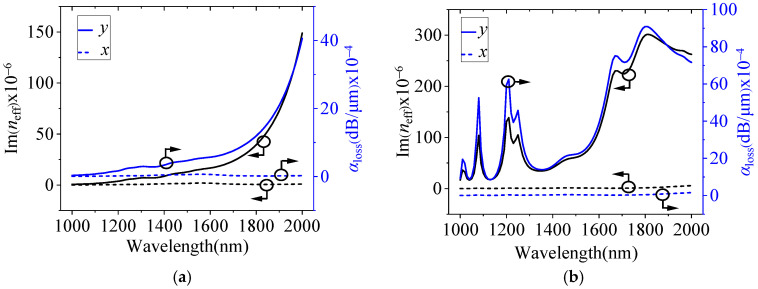
The imaginary part of the effective refractive index and the loss coefficient of the x- and y-polarization modes as a function of wavelength, (**a**) unfilled with MF and (**b**) filled with MF.

**Figure 4:**
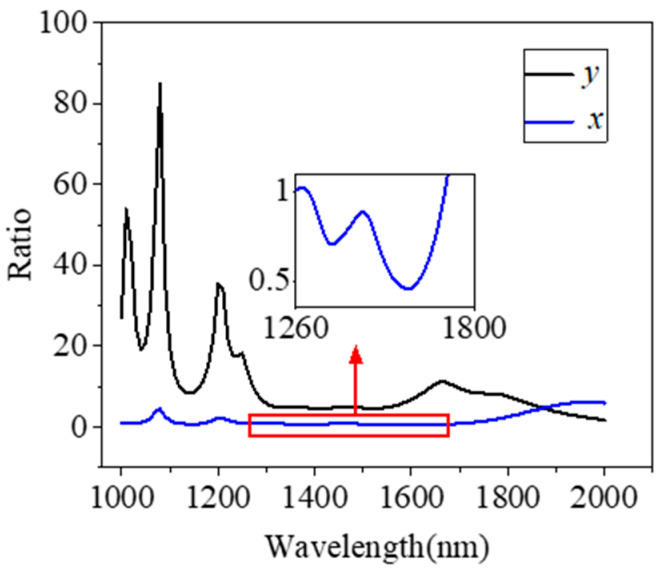
Ratio of each polarization loss versus wavelength when filled with MF and unfilled.

To analyze whether the MF filling has an enhancement effect at different wavelengths, we scanned the imaginary part of the effective refractive index Im(*n*_eff_)) and the loss coefficient *α* in the wavelength range of 1000–2000 nm. Figure 3a,b show the variations in Im(*n*_eff_) and *α* with the wavelength for the x- and y-polarization modes, respectively, both filled and unfilled with MF. In Figure 3, the solid line represents the loss coefficient and the imaginary part of the effective refractive index for the y-polarization mode, and the dotted line denotes the x-polarization mode. Figure 3a depicts the results with unfilled MF; Im(*n*_eff_) and *α* of the y-polarization mode are always larger than that of the x-polarization mode, and the difference rises with the increase in wavelength. Figure 3b indicates that, after filling with MF, Im(*n*_eff_) and *α* of the x-polarization mode remain almost unchanged, while Im(*n*_eff_) and *α* of the y-polarization mode increase significantly. Figure 4 bewrites the variation in each polarization loss ratio with the wavelength when filled with MF and unfilled; the black line is the y-polarization mode, and the blue line represents the x-polarization mode. As can be seen from Figure 4, the loss coefficient of the y-polarization mode after filling is several times that of the unfilled mode, and the highest point reaches 84.9 times, of which the minimum multiple is 4.7 in the communication band of 1260–1675 nm. The inset in Figure 4 is a magnified view of the x-polarization mode in the band between 1260 and 1675 nm. It can be seen from the illustration that after filling with MF, the loss coefficient of the x-polarization mode in the communication band is 0.45 to 1 times that before filling, which means that the loss coefficient of the x-polarization mode is reduced after filling with MF. In conclusion, the performance of the polarization filter can be enhanced with this method.

In the process of designing PCF polarization filters, the impact of the structural parameters on the polarization mode loss coefficient needs to be considered. The structural parameters mainly include the PCF hole spacing *Λ*, the duty cycle *d*/*Λ*, the outer diameter of the gold film air holes, and the gold film thickness *s*. The purpose of this paper is to design a low-loss polarization filter that can be fused with ordinary single-mode optical fibers, and to ensure that the PCF can transmit as single-mode without cutoff, so the hole spacing is 5.25 µm and the duty cycle is 0.44. Thus, we only need to analyze the influence of the outer diameter and thickness of the air holes’ deposited gold film on the loss coefficients of the different polarization modes.

**Figure 5:**
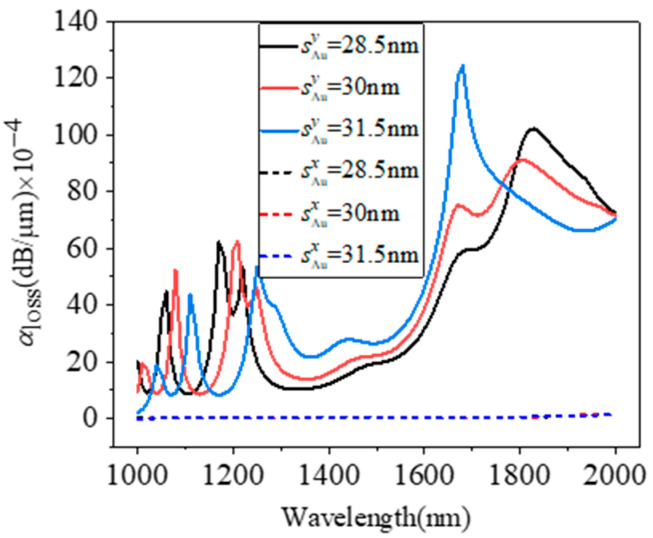
Polarization loss versus wavelength with different *s* of 28.5 nm, 30 nm, and 31.5 nm.

Figure 5 depicts the loss coefficients of the two polarization modes as a function of wavelength when *s* is 28.5 nm, 30 nm, and 31.5 nm. The variation in *s* has almost no effect on the loss coefficient of the x-polarization mode, but has a greater impact on the y-polarization mode. As *s* rises, the peaks move closer to the center, and the value between the two peaks on the right also increases. As shown in Figure 5, at the gold film thickness of 28.5 nm, the peaks are 1060 nm, 1170 nm, and 1830 nm, and the loss coefficients are 44.6 dB/cm, 62.1 dB/cm, and 102.1 dB/cm, respectively. When the thickness is 30 nm, the peaks are 1080 nm, 1210 nm, and 1810 nm, and the loss coefficients are 52.5 dB/cm, 62.4 dB/cm, and 90.9 dB/cm, respectively. When the thickness is 31.5 nm, the peaks are 1100 nm, 1250 nm, and 1680 nm, and the loss coefficients are 43.6 dB/cm, 53.6 dB/cm, and 124.4 dB/cm, respectively. So, to improve the polarization loss coefficient in the communication band, the thickness of the gold film is kept within a positive tolerance as much as possible.

**Figure 6:**
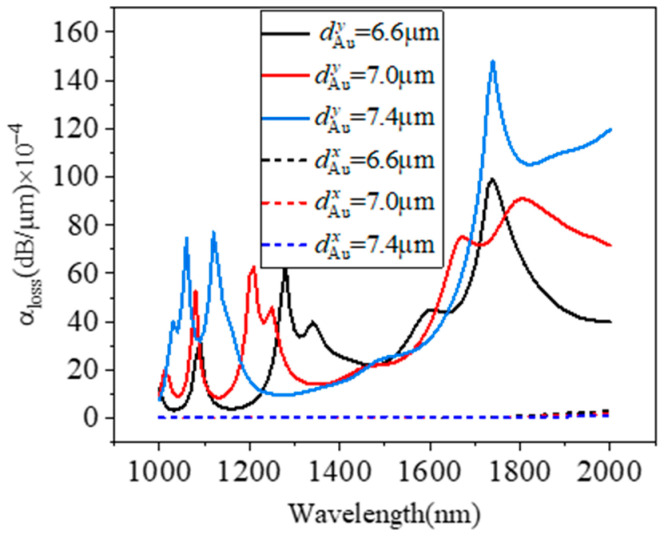
Polarization loss versus wavelength with different *d*_Au_, 6.6 µm, 7 µm, and 7.4 µm.

Figure 6 shows the loss coefficients of the two polarization modes as a function of wavelength when $d_{\mathrm{Au}}$ is 6.6 µm, 7 µm, and 7.4 µm. The variation in $d_{\mathrm{Au}}$ has almost no effect on the loss coefficient of the x-polarization mode, but affects the y-polarization mode. As shown in Figure 6, when $d_{\mathrm{Au}}=6.6\;\mu m$, the peaks are 1090 nm, 1280 nm, and 1740 nm, and the loss coefficients are 32.2 dB/cm, 63.7 dB/cm, and 99.3 dB/cm, respectively. When $d_{\mathrm{Au}}=7$ μm, the peaks are 1080 nm, 1210 nm, and 1810 nm, and the loss coefficients are 52.5 dB/cm, 62.4 dB/cm, and 90.9 dB/cm, respectively. When $d_{\mathrm{Au}}=7.4$ μm, the peaks are 1060 nm, 1210 nm, and 1800 nm, and the loss coefficients are 74.5 dB/cm, 77 dB/cm, and 148.2 dB/cm, respectively. Therefore, when manufacturing the device, the aperture should be kept to a negative tolerance as much as possible.

**Figure 7:**
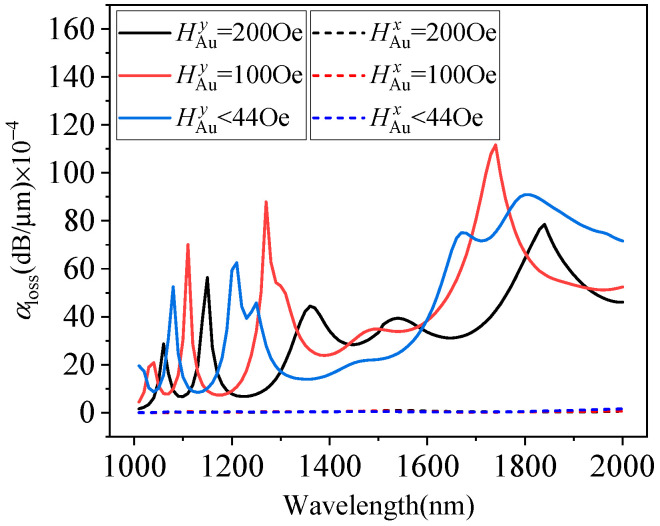
Polarization loss versus wavelength with different magnetic field intensities.

According to Formula (2), the refractive index of the MF can be tuned by changing the strength of the external magnetic field. Therefore, in addition to improving the loss coefficient of the y-polarization mode, the addition of the MF can also optimize the loss coefficient of the y-polarization mode by changing the magnetic field intensity. Figure 7 shows the variation in the x- and y-polarization mode loss coefficients with the wavelength when the magnetic field strength is less than $H_{c}$, and $H_{c}$ is 100 Oe and 200 Oe. When *H* exceeds $H_{c}$, the refractive index of the MF will be affected by the magnetic field strength, which will affect the loss of the y-polarization mode, but will hardly affect the loss of the x-polarization mode. When H<44 Oe, the peaks are 1080 nm, 1210 nm, and 1810 nm, and the loss coefficients are 52.5 dB/cm, 62.4 dB/cm, and 90.9 dB/cm, respectively. When H=100 Oe, the peaks are 1110 nm, 1270 nm, and 1740 nm, and the loss coefficients are 70.1 dB/cm, 87.9 dB/cm, and 111.7 dB/cm, respectively. When H=200 Oe, the peaks are 1150 nm, 1360 nm, and 1850 nm, and the loss coefficients are 56.3 dB/cm, 44.4 dB/cm, and 78.5 dB/cm, respectively. From the above analysis, it can be seen that the position of the polarization mode loss peak can be adjusted by changing the magnetic field strength.

**Figure 8:**
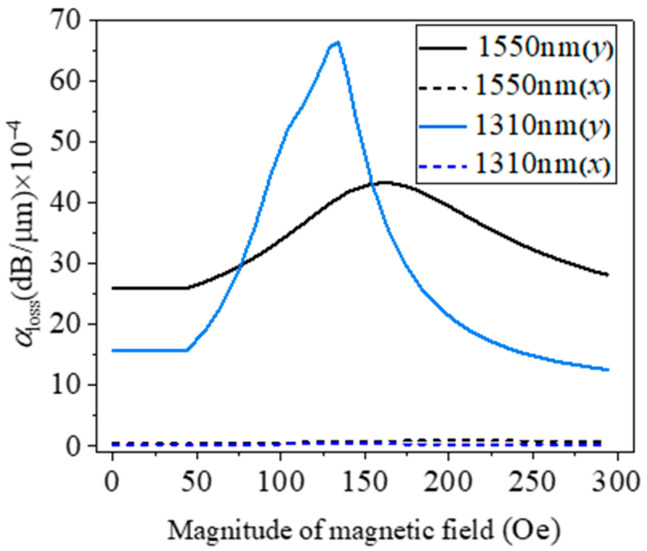
Polarization loss versus magnetic field intensity at different wavelengths, 1310 nm and 1550 nm.

Taking the wavelengths of 1310 nm and 1550 nm as examples, the influence of the magnetic field intensity, varying in the range of 0~300 Oe, on the loss coefficients of the x- and y-polarization modes is analyzed, as shown in Figure 8. The variation in *H* has a greater impact on the y-polarization mode, but has little impact on the x-polarization mode. The y-polarization loss can be enhanced by the bias magnetic field from 15.78 dB/cm to 66.4 dB/cm when *H* increases from 0 to 134 Oe at the wavelength of 1310 nm. The y-polarization loss can be improved by the bias magnetic field from 25.9 dB/cm to 43.4 dB/cm when *H* increases from 0 to 164 Oe at the wavelength of 1550 nm. Therefore, by adjusting the external magnetic field strength, the polarization loss coefficient of a specific wavelength can be optimized.

**Figure 9:**
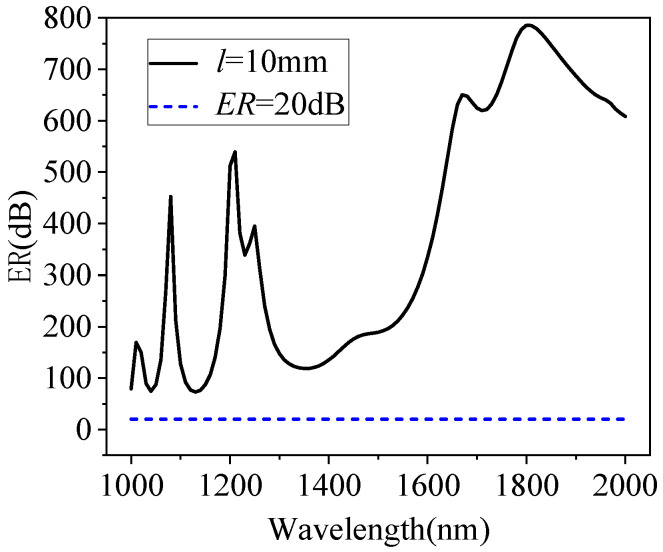
ER versus wavelength with *l* = 10 mm.

**Figure 10:**
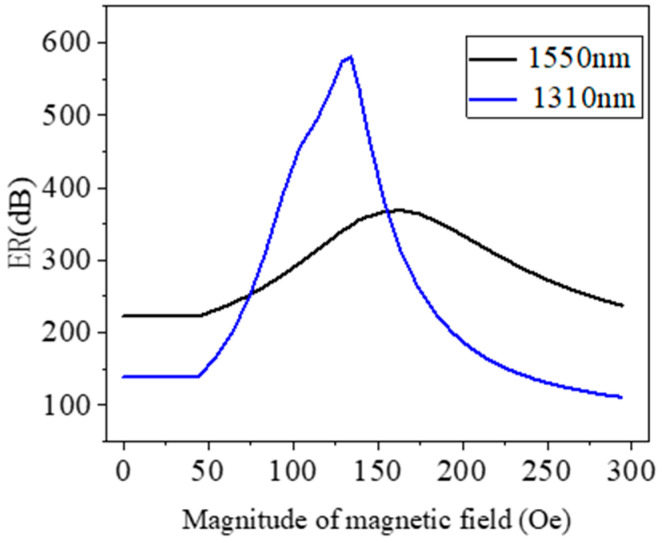
ER versus magnetic field intensities at different wavelengths, 1310 nm and 1550 nm.

For the optical fiber polarization filter, the ER is a key parameter to measure its performance, and it is calculated using Formula (4). Figure 9 demonstrates the variation in the ER with the wavelength, with *l* = 10 mm. The black line in the figure shows the variation in the ER with the wavelength after filling with MF, and the blue dotted line is the reference line, with ER = 20 dB. Figure 10 describes the variations in the ER at the wavelengths of 1310 nm and 1550 nm with the magnetic field intensity *H* when the device length is 10 mm. The ER can be enhanced by the magnetic field from 139.14 dB to 580.3 dB when *H* increases from 0 to 134 Oe at the wavelength of 1310 nm. The y-polarization loss can be improved by the bias magnetic field from 222.72 dB to 368.7 dB when *H* increases from 0 to 164 Oe at the wavelength of 1550 nm. It can be seen from the changes in the key parameter of the ER that the filling of MF not only improves the performance of the polarization filter, but also makes it tunable.

To further evaluate the performance of the proposed optical fiber filter, Table 1 supplies a comparison with some previous works using different structures. It can be seen that reducing the fiber core diameter can greatly improve the performance of the fiber filter, but this greatly increases the splicing loss with single-mode fibers. Therefore, it is necessary to find ways to improve the filter performance without reducing the fiber core diameter. The introduction of 2D materials in the literature [28] effectively enhanced the SPR effect, but the method proposed in this article achieves better results.

## 4. Conclusions

In conclusion, we have conducted a detailed study of a new way of filling the air holes of a PCF, coated with gold film, with MF to enhance filter performance without reducing the fiber core diameter. The numerical analysis results show that this method can effectively enhance the loss coefficient of the y-polarization mode and reduce the loss coefficient of the x-polarization mode. Based on this, we designed a polarization filter with the same diameter as the single-mode optical fiber. The results indicate that the extinction ratio of the filter in the communication band is much greater than 20 dB, with a length of 10 mm. Moreover, the filter can adjust the extinction ratio at different wavelengths through the magnetic field to achieve the optimal state.

## Figures and Tables

**Table 1:** Analogy between suggested design and some prior works.

References	D(µm)	Maximum Loss (dB/cm)	Material Composition	Tuning Method
[23]	2.5	946.63	Au	-
[36]	2.8	2138.34	Au	-
[37]	3.9	1097.94	Au	-
[38]	3.6 × 3.9	265.04	Au	-
[31]	8.2	39	2D + Au	Voltage
This work	8.2	90.9	MF + Au	Magnetic field

## Data Availability

Data is contained within the article.
